# Current Trends and Future Challenges in Transcatheter Aortic Valve Replacement: Utility of Cardiac Computed Tomography Angiography

**DOI:** 10.3390/jcm14072474

**Published:** 2025-04-04

**Authors:** Georgios Tzimas, David Meier, Eirini Beneki, Panagiotis Antiochos, Denise Auberson, Simon Leboube, Henri Lu, Quentin Liabot, Aurelia Zimmerli, Adil Salihu, Pierre Monney, Olivier Muller, Mariama Akodad, Stephane Fournier

**Affiliations:** 1 Department of Cardiology, Lausanne University Hospital, University of Lausanne, 1005 Lausanne, Switzerland; david.meier@chuv.ch (D.M.); eirini.beneki@chuv.ch (E.B.); panagiotis.antiochos@chuv.ch (P.A.); denise.auberson@chuv.ch (D.A.); simon.leboube@chuv.ch (S.L.); henri.lu@chuv.ch (H.L.); quentin.liabot@chuv.ch (Q.L.); aurelia.zimmerli@chuv.ch (A.Z.); adil.salihu@chuv.ch (A.S.); pierre.monney@chuv.ch (P.M.); olivier.muller@chuv.ch (O.M.); 2 Institut Cardiovasculaire Paris-Sud, Hôpital Privé Jacques Cartier, Ramsay-Santé, 91100 Massy, France; akodadmyriam@gmail.com

**Keywords:** transcatheter aortic valve replacement, cardiac computed tomography angiography

## Abstract

Transcatheter aortic valve replacement (TAVR) has revolutionized the treatment of aortic stenosis. As TAVR continues to evolve, precise pre-procedural planning and imaging have become increasingly critical. While transthoracic echocardiography remains indispensable for assessing the severity of aortic stenosis, cardiac computed tomography angiography (CCTA) has emerged as the benchmark imaging modality for pre-procedural planning for TAVR. CCTA provides detailed anatomical information essential for patient selection, procedural success, and the mitigation of complications. This review aims to equip practitioners with the knowledge to effectively integrate CCTA into the TAVR workflow, ensuring a systematic approach to patient evaluation and procedural planning while addressing future challenges in the field.

## 1. Introduction

Over the past two decades, transcatheter aortic valve replacement (TAVR) has emerged as a safe and effective treatment strategy for patients with severe symptomatic aortic valve diseases across all surgical risk groups [1,2,3,4]. Advances in this procedure have underscored the critical role of pre-procedural imaging in optimizing patient selection, guiding procedural planning, and improving clinical outcomes. While transthoracic echocardiography remains essential for assessing the severity of aortic stenosis, computed tomography (CT) has become the gold standard for anatomical evaluation in TAVR planning. Since the early days of TAVR, CCTA has progressively evolved from an adjunct imaging tool to an indispensable modality, owing to its ability to provide comprehensive anatomical assessments that enhance procedural safety and outcomes.

Cardiac CT angiography (CCTA) provides vital information for procedural success, including detailed characterization of the aortic root, coronary ostia height, and vascular access routes. Additionally, it enables precise fluoroscopic angle selection for valve deployment and plays a pivotal role in assessing valvular calcification, particularly in complex cases such as low-flow, low-gradient aortic stenosis. CCTA is also essential to identify patients with increased anatomical risk for coronary artery occlusion in valve-in-valve (ViV) procedures. These capabilities not only enhance procedural accuracy but also reduce potential complications, reinforcing the essential role of CCTA in contemporary TAVR workflows. This review examines the integral role of CCTA in TAVR, emphasizing a structured approach to patient evaluation and procedural planning while addressing future challenges in the field.

## 2. Aortic Valve Calcium Score

Precise identification of severe aortic stenosis is essential for determining the appropriate timing and approach to treatment. However, evaluating clinically significant aortic stenosis can be challenging, particularly when standard echocardiographic parameters—such as mean transvalvular gradient, peak aortic velocity, and aortic valve area—yield discordant findings. These inconsistencies, which may also be observed in invasive hemodynamic assessments, affect approximately 25% of patients, especially those with reduced left ventricular ejection fraction. A well-established and increasingly recognized tool for the evaluation of aortic stenosis severity is the aortic valve calcium score, which is determined using the Agatston method with non-contrast CT. The burden of aortic valve calcification as assessed by CT has been demonstrated to aid in the stratification of AS severity in cases where conventional echocardiographic metrics fail to yield a conclusive diagnosis [5]. According to current guidelines, assessment of the aortic valve calcium score is advised for patients with suspected severe aortic stenosis who exhibit low-gradient findings (peak systolic velocity of <4 m/sec, mean gradient of <40 mm Hg) and reduced stroke volume index (<35 mL/m^2^). This evaluation is particularly recommended in cases where left ventricular ejection fraction (LVEF) is reduced to below 50% without evidence of flow reserve on dobutamine stress echocardiography or when LVEF remains preserved at ≥50%. Specific Agatston unit thresholds for severe AS classification have been established as follows: value of >3000 for men and value of >1600 for women = highly likely, value of >2000 for men and value of >1200–1300 for women = likely, and value of <1600 for men and value of <800 for women = unlikely [6,7].

Several semiautomated CT-based methods are available for quantifying aortic valve calcification. The Agatston score applies a CT attenuation threshold of ≥130 HU to identify calcified regions. Each lesion is assigned a weighting factor based on its maximum attenuation: 1 for 130–199 HU, 2 for 200–299 HU, 3 for 300–399 HU, and 4 for ≥400 HU. The final Agatston score is derived by multiplying the weighting factor by the lesion area and summing the values for all calcified regions. Accurate evaluation of aortic valve calcification using CT remains a challenging endeavor, necessitating meticulous delineation of valvular from non-valvular calcifications. Both leaflet and annular calcifications must be included while systematically excluding non-valvular calcifications originating from the left ventricular outflow tract, aortic sinuses, coronary arteries, and mitral annulus. The standard approach for quantification involves performing measurements on axial CT slices, ensuring that calcium tracking is conducted in a continuous manner on adjacent axial images in both cranial and caudal directions. Nevertheless, to enhance visualization and ensure precise identification of valvular calcium, it is advantageous to assess the aortic valve in multiple planes, including the aortic annular plane. Despite these complexities, the reproducibility of aortic valve calcium score measurements is well-documented, with excellent intraobserver and interobserver agreement reported in the literature [8].

Given the inherent challenges associated with aortic valve calcium quantification, some investigators have explored the potential utility of reorienting and reconstructing CT images into a short-axis view of the aortic valve. This approach facilitates improved differentiation of valvular from non-valvular calcifications and enables the assessment of calcium burden on a per-leaflet basis. Although the short-axis view offers distinct advantages in distinguishing valvular from extravalvular calcifications, it should not be utilized for definitive interpretation. This is due to the fact that reorientation of CT images alters the aortic valve calcium score, leading to a systematic reduction in the measured calcium burden compared to standard axial views [9].

## 3. Aortic Valve Assessment

Traditionally, the evaluation of aortic valve morphology has been performed using transthoracic echocardiography. However, CCTA offers complementary anatomical detail, enhancing the accuracy of preprocedural planning for TAVR. A comprehensive assessment of the aortic valve encompasses a detailed description of the valve morphology (tricuspid vs. bicuspid), the number of cusps, their thickness, and the extent of calcification. This assessment is essential, as (i) outcomes of TAVR in bicuspid aortic stenosis are influenced by valve morphology [10] and (ii) the transcatheter heart valve (THV) is implanted at the level of the aortic annulus and cusps, where anatomical characteristics play a key role in procedural success [11].

The morphology of bicuspid aortic valve is defined based on the presence of a raphe and the number of commissures, resulting in three broad morphologies: (i) tricommissural, often termed ’functional’ bicuspid aortic valve (not part of Sievers classification) with a residual commissure; (ii) bicommissural raphe type (equivalent to Sievers type 1); and (iii) bicommissural non-raphe type (equivalent to Sievers type 0) [12]. The presence of a raphe plays a significant role, particularly if the raphe is calcified, as it may impact THV expansion. Consequently, the degree of raphe calcification should always be analyzed, as severe calcification is associated with an increased risk of paravalvular regurgitation and a higher risk of annular injury [13]. Sizing at the level of the raphe in the bicuspid aortic valve as well as 4 mm above the annulus plane in order to provide a better understanding of the bicuspid aortic valve morphology may be interesting [14]. The presence or absence of a third commissure and the intercommissural distance may also affect THV apposition, further increasing the risk of paravalvular leak [12].

## 4. Aortic Root Assessment

### Aortic Annulus Assessment

The aortic annulus in a tricuspid aortic valve is defined by the nadir of the aortic cusps. Achieving an accurate assessment of the aortic annular plane necessitates the precise identification of each of these three points. Given the significant differences in the area and perimeter of the aortic annulus between systole and diastole [15,16], it is essential to identify the reconstruction phase with the largest annular dimensions and satisfactory image quality, as this is crucial for THV sizing. In general, aortic annular dimensions are larger in mid-systole; however, in cases of inverse annular dynamism associated with septal hypertrophy, the maximal annular dimensions may occur during diastole. Determining the annular dimensions and valve morphology can be particularly challenging in patients with bicuspid aortic valve. In a Sievers type 0 bicuspid aortic valve, the absence of a third commissure results in only two hinge points for defining the annular plane, complicating precise annular dimension measurements. The identification of the aortic annular plane can be performed using different CT techniques [17].

During the pre-procedural diagnostic assessment, a thorough evaluation of annular and subannular calcifications is essential, including a detailed characterization of calcium morphology, the number of nodules, their configuration, and their spatial distribution. Particular attention should be given to large, protruding nodules, as they are associated with an increased risk of annular rupture, especially when using balloon-expandable THVs [18].

## 5. Coronary Ostial Height and Sinus of Valsalva Assessment

Coronary occlusion, though rare, occurs in approximately 0.66% of TAVR procedures and is associated with a high 30-day mortality rate (up to 40.9%), even when rescue revascularization is attempted [19,20]. The risk of coronary obstruction is higher in ViV TAVR (2.3%) [21]; however, the absolute incidence remains greater in native aortic stenosis due to the higher procedural volume. This complication arises when the native valve leaflets are displaced toward the coronary ostia (coronary ostial obstruction), most commonly affecting the left main coronary artery, or the sinotubular junction (sinus sequestration). Acute hemodynamic collapse is the predominant clinical presentation, although delayed onset has also been reported.

**Figure 1:**
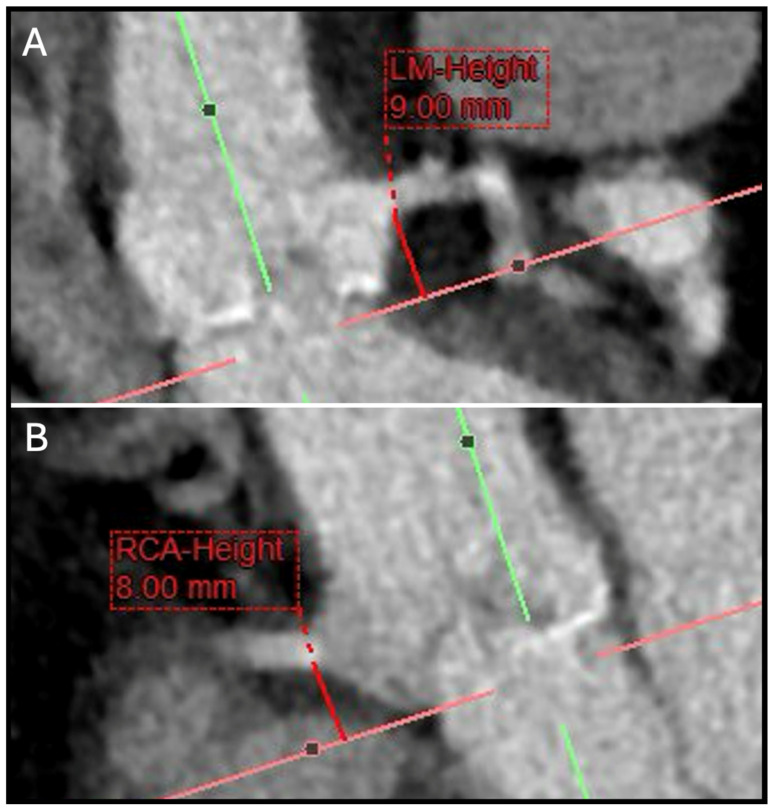
Pre-TAVR CT work-up with two examples of coronary ostia height (**A**) LM-Height (**B**) RCA-Height. The coronary height should by assessed by measuring the distance from the aortic annulus plane to the lower-level margin of the coronary ostia. CT: computed tomography; LM: left main; RCA: right coronary artery;TAVR: transcatheter aortic valve replacement.

Assessing the risk of coronary occlusion requires a comprehensive evaluation of the spatial relationship between the native aortic leaflets, the coronary arteries, the sinotubular junction, and the dimensions of the intended THV. CCTA is the gold standard for pre-procedural risk assessment. Low coronary ostial height (<12 mm) and a sinus of Valsalva diameter of <30 mm are recognized as risk factors (Figure 1); however, these measurements lack absolute specificity and should not be interpreted in isolation. Instead, coronary ostial height should be considered in the broader context of annular dimensions, overall aortic root anatomy, and the anticipated THV size. A novel CT-based multivariate prediction model, which can be routinely implemented in clinical practice, has been developed to further assess the risk of coronary artery obstruction following TAVR in patients with native aortic stenosis. This model, which incorporates cusp height exceeding coronary ostial height, a virtual valve-to-coronary (VTC) distance of ≤4 mm, or a culprit leaflet calcium volume >600 mm^3^, has demonstrated strong predictive performance in identifying patients at risk of this complication [22].

## 6. CT Fluoroscopic Angulation

In the context of TAVR procedural planning, identifying a coplanar fluoroscopic view of the aortic valve is recommended, typically achieved through CCTA. Traditionally, the three-cusp view has been employed, in which the three aortic cusps are aligned, with the right coronary cusp positioned centrally between the non-coronary and left coronary cusps [23]. However, the left/right cusp overlap view, which involves a coplanar projection overlapping the right and left coronary cusps, has gained increasing favor, particularly for the deployment of self-expanding THVs [24,25]. This approach offers several advantages for self-expanding devices, including minimization of catheter parallax, reduction of the distance between the non-coronary and left coronary cusps, improved visualization of the membranous septum, and enhanced precision in implantation depth and positioning. Although it has been mostly studied for self-expanding valves where pacemaker rate was particularly an issue, it also allows a more precise positioning for balloon-expandable valves. In addition, if the identified fluoroscopic projections require cranial or caudal angulations exceeding 35°, alternative angulations should be considered due to the physical constraints of the C-arm. Of note, it is important to recognize that CT-predicted angulations remain valid only if the patient’s chest position during CT acquisition is replicated during the procedure.

## 7. Conduction Abnormalities

Severe calcification within the sub-annular landing zone may lead to compression and injury of the conduction system by the THV, potentially resulting in atrioventricular block and necessitating pacemaker implantation [26,27]. A lower implantation depth of the THV has been consistently associated with an increased risk of left bundle branch block, irrespective of whether a balloon-expandable or self-expanding prosthesis is used [28,29]. Notably, optimizing implantation depth during TAVR has been shown to significantly reduce both mortality rates and the need for permanent pacemaker placement. Furthermore, a short membranous septum, defined as being of a length of less than 8 mm, has been identified as a predictor of conduction disturbances following TAVR [30]. The length of the membranous septum can be accurately assessed in the coronal plane, specifically at its longest measurement between the annular level and the muscular septum.

## 8. THV Sizing

CCTA remains the non-invasive imaging gold standard for annular sizing and THV selection. In clinical practice, self-expanding valves are typically sized based on annular perimeter measurements, whereas balloon-expandable valves are primarily sized according to annular area. Sizing methodologies relying on manually measured short- and long-axis diameters have demonstrated lower reproducibility and are now considered outdated [31].

Annular oversizing is determined using the following formula: oversizing [%] = (THV nominal measurement/annular measurement −1) × 100. However, it is crucial to recognize that the calculated oversizing percentage is highly dependent on the specific annular measurement used [32]. In the case of a perfectly circular annulus, area-based oversizing is approximately twice the perimeter-based oversizing, regardless of whether the measurement is derived from area-based or perimeter-based diameters. However, in non-circular annular geometries, oversizing based on the perimeter-derived diameter tends to be lower than that based on the area-derived diameter. Importantly, excessive oversizing, particularly beyond 20%, is associated with an increased risk of aortic annular rupture [33].

Beyond annular sizing, CCTA plays a crucial role in TAVR by providing precise measurements of annular dimensions and detailed assessment of annular and subannular calcifications. When calcifications extend into the lumen, they significantly elevate the risk of annular rupture and paravalvular regurgitation. In particular, calcifications protruding into the landing zone beneath the non-coronary cusp have been strongly linked to annular injury, with this risk further exacerbated in the presence of aggressive annular oversizing [34].

## 9. Valve-in-Valve TAVR

The CCTA assessment for ViV TAVR procedures should adhere to the same acquisition CT protocol as standard TAVR. Identifying the implanted prosthetic heart valve is essential for evaluating the feasibility of ViV TAVR and optimizing procedural planning. The manufacturer, model, and size of the previously implanted valve can typically be retrieved from the original operative report. However, if this information is not available in the patient’s medical records, CT imaging can assist in estimating the valve type [35]. Reference charts are available to approximate valve sizes, though they may not cover the full spectrum of THVs or previously implanted surgical heart valves (SHV) [35,36,37]. Performing ViV TAVR presents unique pre-procedural challenges, as prosthetic valves can be surgical (stented, sutureless, or stentless) or transcatheter, each with distinct characteristics that may require a tailored approach to optimize outcomes.

### 9.1. TAVR in SHV

In stented or sutureless surgical heart valves (SHVs), the bioprosthetic leaflets remain open after THV implantation, creating a cylindrical structure that may impede blood flow through the struts of the new valve. If the displaced bioprosthetic leaflets extend above the coronary ostia, assessing the aortic sinus dimensions becomes essential to ensure adequate coronary perfusion.

**Figure 2:**
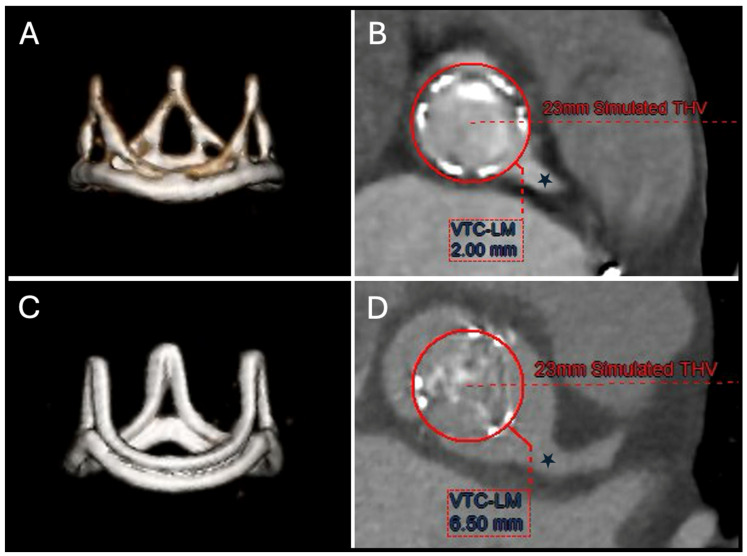
Pre-ViV TAVR CT work-up with two examples of THV simulation. (**A**) Volume rendered images showing an SHV (Trifecta, St Jude, 21 mm). (**B**) According to the Valve-in-Valve app, the VTC-to-LM (black star) distance after simulating a 23-mm simulated THV is measured at 2 mm. (**C**) Volume rendered images showing an SHV (Perimount, Magna Ease 3300 TFX, 21 mm). (**D**) According to the Valve-in-Valve app, the VTC-to-LM (black star) distance after simulating a 23 mm simulated THV is measured at 6.5 mm. LM: left main; SHV: surgical heart valve; TAVR: transcatheter aortic valve replacement; THV: transcatheter heart valve; VTC: valve-to-coronary distance; ViV: valve-in-valve.

A proposed method for evaluating this risk of coronary obstruction after ViV TAVR in SHV is the virtual THV-to-coronary (VTC) distance (Figure 2), which estimates the distance between the new THV frame and the coronary ostia. A VTC distance of <4 mm is a strong independent predictor of coronary obstruction in ViV TAVR [21]. Compared to native valve TAVR, the risk of coronary obstruction is 3- to 4-fold higher in ViV cases, with the VIVID Registry initially reporting an incidence of 3.5%. However, this may be underestimated due to incomplete obstruction or patent bypass grafts mitigating the effect. For cases with a high risk of coronary obstruction due to a small VTC distance, leaflet modification techniques like BASILICA (Bioprosthetic Aortic Scallop Intentional Laceration to prevent Iatrogenic Coronary Artery obstruction) can be used to lacerate the interfering SHV leaflet before TAVR [38].

Another major concern in TAVR within SHVs is the risk of coronary obstruction due to sinus of Valsalva sequestration. If the displaced bioprosthetic leaflets extend above the sinotubular junction (STJ) and come into close proximity or direct contact with it, coronary flow may be compromised following THV implantation. A narrow virtual THV-to-sinotubular junction (VTSTJ) distance (<2 mm) has been associated with an increased risk of sinus sequestration, likely due to reduced diastolic filling of the sinuses of Valsalva [39,40]. The same CT-based method used for VTC distance assessment is employed to estimate VTSTJ distance.

Despite meticulous pre-procedural CT assessment, some data suggest that there is a consistent underestimation of VTC and VTSTJ distances when comparing pre-procedural virtual measurements to post-procedural TAVR CT findings [41]. This discrepancy is often attributed to routine THV conical underexpansion of the THV stent frame with flaring toward the outflow.

Of note, in patients with stented or sutureless SHVs with radiopaque scaffolds, a non-contrast CT scan may be sufficient to evaluate the risk of coronary obstruction, as the coronary ostia can often be identified based on the surrounding epicardial fat. However, in cases involving stentless SHVs, the assessment should follow the same approach as for native aortic stenosis. This requires a standard TAVR CT protocol with contrast enhancement, including the measurement and documentation of sinus dimensions and coronary heights, as previously described for native aortic stenosis.

It is essential to acknowledge the limitations of relying solely on two-dimensional anatomical measurements, such as VTC or VTSTJ, which do not fully account for the complex three-dimensional interaction between the THV and native cardiac structures or their combined effect on coronary blood flow dynamics. CT-guided computational modeling offers a promising approach to refine risk assessment, enabling a more personalized and precise evaluation that enhances patient selection and procedural planning in transcatheter interventions [42].

### 9.2. TAVR in TAVR

The same proposed CT method for assessing the risk of coronary obstruction and sinus sequestration by estimating the VTC and VTSTJ distances, respectively, should be applied in redo-TAVR cases. However, a crucial aspect of planning for redo-TAVR is understanding the interaction between the existing THV, the coronary ostia, and the sinotubular junction, particularly when implanting a second THV. It is essential to distinguish between two key concepts: neoskirt and functional neoskirt.

The term neoskirt refers to the newly formed tubular structure created when the leaflets of the original THV are displaced upward by the implantation of a second THV. The height of this structure depends on the specific combination of THVs used and can vary considerably. The shortest neoskirt is typically achieved when the index THV is a balloon-expandable valve since the neoskirt will generally never extend beyond the top of its relatively short frame unless the second THV is implanted very high. In contrast, performing redo-TAVR inside a self-expanding platform can result in the neoskirt extending largely above the coronary ostia and even beyond the STJ. Recognizing these variations is essential for optimizing redo-TAVR planning and procedural outcomes [43,44].

The functional neoskirt is the most clinically relevant, as it represents the portion of the neoskirt extending above the annular plane, which is directly influenced by the implantation depth of the original THV. While the total neoskirt height remains constant for a given THV combination, a deeper implantation of the initial valve results in a shorter functional neoskirt. This distinction highlights the importance of precise depth control during THV deployment to optimize functional neoskirt height and improve procedural outcomes in redo-TAVR.

Assessing functional neoskirt height in relation to coronary ostia positioning enables a straightforward evaluation using CCTA simulation to determine whether the risk plane extends above the coronary arteries. If the predicted neoskirt height remains below the coronary level, the likelihood of coronary obstruction is low. Leaflet modification techniques, such as BASILICA, may serve as mitigation strategies; however, their success depends on proper commissural alignment of the index valve. The lower efficacy of the BASILICA procedure may arise in two specific scenarios: (i) in cases of significant coronary eccentricity, where leaflet splay does not occur directly in front of the coronary ostia, potentially compromising coronary protection, and (ii) when performing BASILICA in a previously implanted THV that is misaligned with the native coronary arteries, which may reduce the effectiveness of the procedure in preserving coronary perfusion. Emerging THV technologies incorporating alignment markers and taking into consideration coronary artery anatomical variability and intercoronary angle may help standardize commissural alignment in the future.

## 10. Vascular Access

CCTA plays a crucial role in pre-procedural planning for TAVR or ViV TAVR, particularly in assessing vascular access. The iliofemoral arteries are the preferred access route, necessitating a thorough evaluation of vessel caliber, calcification pattern, and tortuosity. Key factors include the minimal luminal diameter, the presence of horseshoe calcifications, and the suitability of the common femoral artery puncture site, as these may impact arterial access and closure device deployment. Of note, in heavily calcified vessels, blooming artifacts and partial volume averaging can lead to an overestimation of plaque burden and an underestimation of the true luminal diameter [45]. Additionally, CCTA can detect high-risk vascular features, such as dissection and complex atheroma, not only in the iliofemoral arteries but also in the aorta, which may influence procedural strategy and outcomes [46]. Of note, given the continuous evolution of delivery systems, it is difficult to provide references to specific current devices and vessel diameter requirements. However, intravascular lithotripsy has made it possible to perform TAVR in patients with severely calcified iliofemoral disease and adequate calcium distribution [47].

When transfemoral access is unsuitable, alternative routes, such as trans-subclavian, trans-axillary, or trans-carotid approaches, may be considered, each with specific anatomic and procedural considerations [48,49]. If these options are not feasible, transcaval access can serve as an alternative in selected expert centers [50,51]. This technique involves delivering the TAVR system into the abdominal aorta via the femoral vein through the inferior vena cava. Therefore, a meticulous evaluation of the aortic wall for calcification-free windows is essential to ensure safe passage from the inferior vena cava into the abdominal aorta.

## 11. Post-TAVR CT Assessment

The current body of evidence supporting the use of post-TAVR cardiac CT remains limited, aside from its established role in the pre-procedural assessment for ViV TAVR. At present, the primary recognized indication is the evaluation of clinical suspicion of leaflet thrombosis. However, emerging data suggest a broader utility, supporting its role in identifying post-procedural complications, detecting valve underexpansion and diagnosing patient-prosthesis mismatch, assessing structural valve degeneration, and evaluating coronary access.

## 12. Hypoattenuating Leaflet Thickening

Leaflet thrombosis in transcatheter aortic valve replacement (TAVR) is characterized by hypoattenuated leaflet thickening (HALT) and restricted leaflet motion, both of which can be identified on post-TAVR CCTA. The reported prevalence of CT-defined HALT following TAVR ranges from 10% to 31%, reflecting its dynamic nature, with onset and resolution occurring along variable timelines [52,53,54,55]. This variability contributes to ongoing uncertainty regarding its clinical implications. Longitudinal analyses of serial post-TAVR CCTA scans have demonstrated substantial heterogeneity in the trajectory of HALT, with a significant proportion of patients exhibiting spontaneous resolution within one year, while others develop HALT despite no evidence of it at 30-day follow-up.

**Figure 3:**
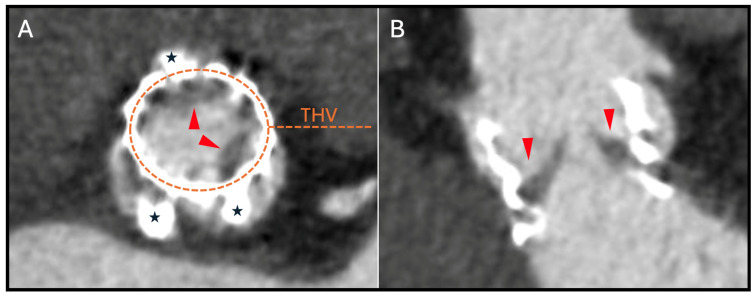
Example of hypoattenuated leaflet thickening in a balloon-expandable device. CT appearance of a THV on CT: (**A**) short axis and (**B**) double oblique transverse plane on a heavily calcified aortic valve (aortic calcification: black stars) with subclinical leaflet thrombosis (red arrowheads), characterized by hypoattenuated leaflet thickening (grade II). CT: computed tomography; THV: transcatheter heart valve.

HALT is identified by the presence of localized leaflet thickening, typically exhibiting a meniscal pattern that is most prominent at the leaflet base and extends toward the tip of the leaflet, particularly on the aortic side (Figure 3). The assessment of HALT severity follows a semiquantitative approach, incorporating both the number of affected leaflets and the degree of thickening. This thickening is stratified into four discrete categories: none and <25%, 25–50%, 50–75%, and >75% involvement [17]. In cases where HALT is detected, a subsequent semiquantitative analysis is performed to assess the presence of reduced leaflet motion, which is visually graded based on the extent of systolic opening restriction. Isolated reduced leaflet motion in the absence of discernible thickening on CCTA is an infrequent finding and should be interpreted with utmost caution to mitigate the potential for unnecessary treatment.

The clinical significance of subclinical HALT remains incompletely defined, with existing observational data suggesting a potential association with increased rates of all-cause mortality, cardiovascular death, ischemic stroke, and heart failure-related hospitalizations [56,57]. Additionally, concerns have been raised regarding the long-term implications of HALT and restricted leaflet motion on THV durability, particularly in relation to the accelerated development of structural valve degeneration. While anticoagulation therapy has demonstrated effectiveness in both the prevention and resolution of subclinical HALT, randomized clinical trials have underscored the associated risks, notably an increased incidence of major bleeding and all-cause mortality, without a significant reduction in stroke events compared to findings for patients managed solely with antiplatelet therapy [58,59,60]. As a result, routine post-TAVR CCTA screening for subclinical HALT is not currently recommended in clinical practice. Instead, targeted CCTA evaluation is reserved for patients presenting with progressive or unexpectedly elevated transvalvular gradients on echocardiography or those exhibiting clinical or imaging findings suggestive of acute thromboembolic events. Further research is warranted to refine clinical decision-making regarding the optimal management of HALT, particularly in cases where extensive leaflet involvement or severe motion restriction is observed, to determine whether a more aggressive therapeutic strategy is justified.

## 13. THV Underexpansion

**Figure 4:**
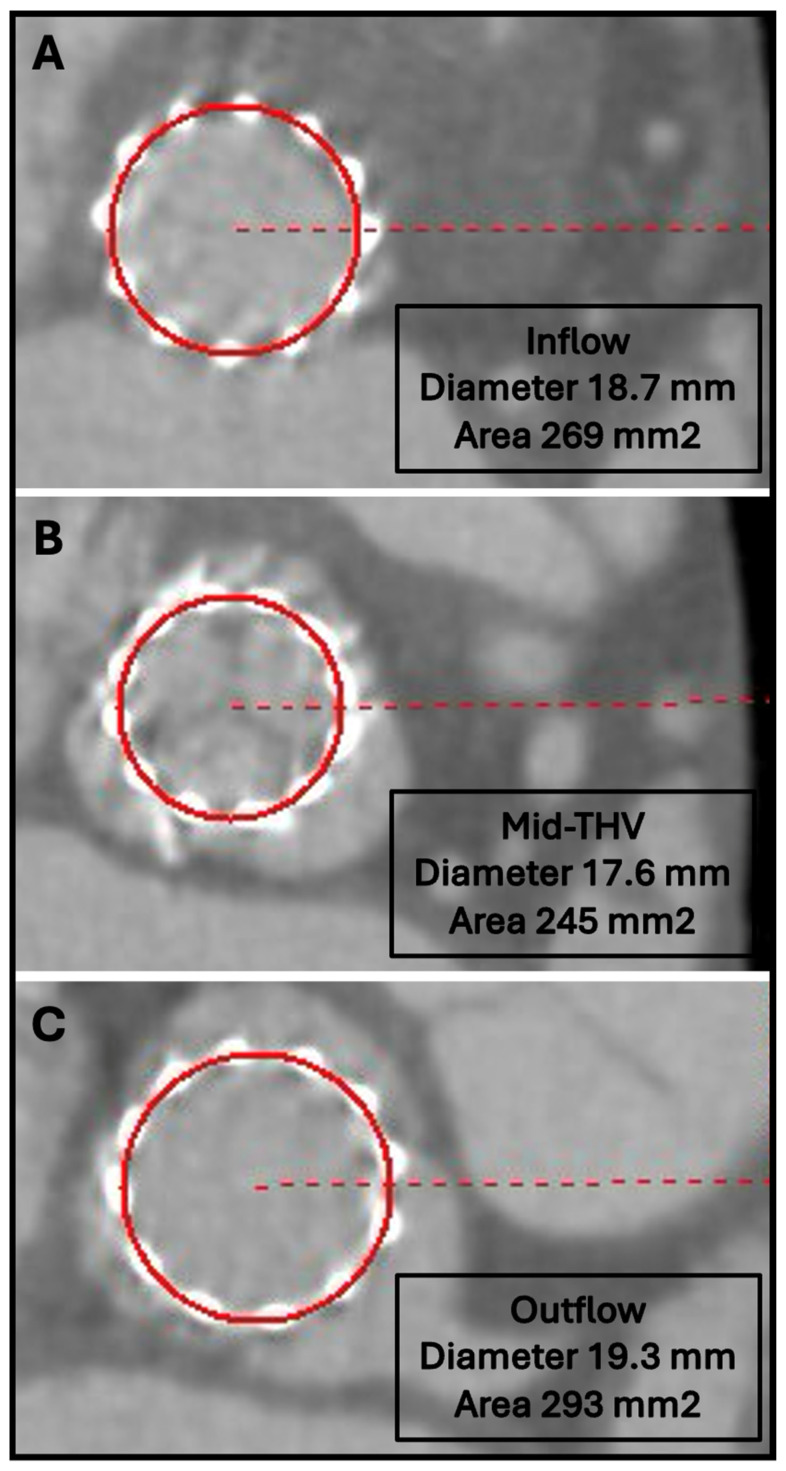
Example of an underexpanded THV (Sapien Ultra 20 mm) after TAVI. (**A**–**C**) Multiplanar reformatted images showing one example of an underexpanded THV (Sapien Ultra 20 mm) after TAVI. Short-axis images aligned across the three different levels (A: Inflow; b: Mid-THV; C: Outflow) showing the different degree of THV expansion.

Suboptimal expansion of THVs is a frequently observed phenomenon following TAVR, with some studies reporting its occurrence in up to 90% of patients receiving balloon-expandable prostheses [57]. This incomplete expansion can have significant hemodynamic and structural consequences, including paravalvular regurgitation, increased transvalvular gradients, and abnormal leaflet motion—commonly referred to as “pin-wheeling”—which has been hypothesized to contribute to excessive leaflet stress. Furthermore, although controversial, underexpansion has been implicated in the development of HALT and is considered a potential precursor to THV dysfunction, with the possibility of accelerating structural valve degeneration and compromising long-term prosthesis durability [57,61,62,63]. Since TAVR has emerged as the predominant therapeutic approach for patients with aortic stenosis across various risk profiles, the early identification and management of THV underexpansion could be of utmost importance to optimize procedural and long-term outcomes. CCTA remains the gold standard imaging modality for post-TAVR assessment of THV expansion, providing detailed insights into stent frame geometry and leaflet configuration. In cases where significant underexpansion is detected, post-TAVR valvuloplasty has been shown to be an effective intervention to improve valve expansion and hemodynamic performance [64]. Of note, contrast administration is not needed to assess THV expansion. A non-contrast full cardiac cycle CT with thin-slice reconstruction (0.5 mm) is recommended for accurately evaluating THV stent frame geometry. Figure 4 illustrates an example of a THV exhibiting suboptimal expansion following TAVR.

## 14. Structural Valve Degeneration

**Figure 5:**
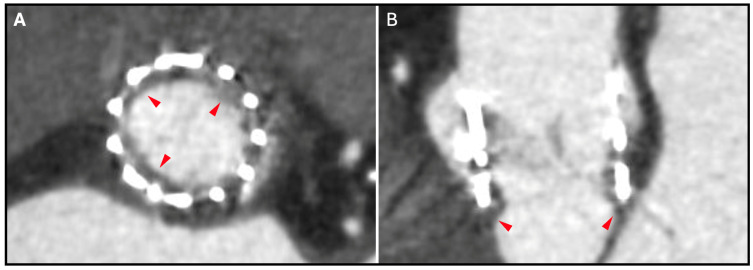
Example of THV pannus. Example of THV pannus (**A**) short axis and (**B**) double oblique transverse plane showing a circular circumferential hypodense tissue (red arrowheads) on the ventricular side at the inflow of the THV suggestive of pannus formation. CT: computed tomography; THV: transcatheter heart valve. CT showing a circular circumferential hypodense tissue (red arrowheads) on the ventricular side at the inflow of the THV (black arrows) suggestive of pannus formation. CT: computed tomography; THV: transcatheter heart valve.

Structural valve degeneration is characterized by alterations in the THV architecture, encompassing leaflet thickening, calcification, tears, structural disruptions, or pannus formation. These pathological changes can result in valvular dysfunction, manifesting as stenosis and/or intra-valvular regurgitation. CCTA provides critical diagnostic insights into the etiology of THV dysfunction by differentiating between thrombus and pannus. Pannus typically appears as a circumferential, hypodense tissue located on the ventricular side of the bioprosthetic valve (Figure 5), whereas thrombus is more commonly found on the aortic side. Additionally, pannus exhibits higher attenuation on CCTA compared to thrombus, aiding in accurate characterization and guiding appropriate management strategies [65]. A non-contrast CCTA can be used to identify the presence of leaflet micro-calcifications as an early sign of THV degeneration.

## 15. Patient–Prosthesis Mismatch

Patient–prosthesis mismatch occurs when the effective orifice area of a THV is disproportionately small relative to the patient’s body size, leading to hemodynamic obstruction despite structurally normal valve function. Patient–prosthesis mismatch is associated with persistently elevated transvalvular pressure gradients, which may negatively impact long-term hemodynamics and clinical outcomes. As PPM does not result from intrinsic prosthetic dysfunction, distinguishing it from structural valve deterioration is essential. Post-TAVR CCTA is a valuable imaging modality for assessing leaflet morphology and motion while aiding in the exclusion of pathological causes of increased gradients, such as THV underexpansion, bioprosthetic leaflet degeneration, thrombus, pannus formation, or, more rarely, endocarditis.

## 16. Coronary Access

As TAVR is increasingly performed in younger patients with longer life expectancies, the likelihood of encountering clinically significant coronary artery disease necessitating percutaneous coronary intervention over time is expected to rise. However, post-TAVR coronary access may be technically challenging due to interactions between the THV structure and the coronary ostia. The complexity of selective coronary cannulation is influenced by multiple anatomical factors, including the position of the commissural posts, the design and extent of the sealing skirt, and the configuration of the stent frame [66,67,68]. These challenges are particularly pronounced in patients receiving self-expanding supra-annular THVs, as compared to intra-annular THVs with shorter frames. The structural characteristics of supra-annular THVs—such as an extended stent frame that may project beyond the sinotubular junction, reduced inter-strut spacing due to smaller stent cells, a more elevated leaflet position, and an asymmetric sealing skirt—can contribute to partial or complete obstruction of the coronary ostia, thereby complicating coronary reaccess [43,69,70].

CCTA plays a critical role in the post-TAVR evaluation of coronary accessibility by identifying anatomical risk factors that may predict procedural difficulties. A key initial assessment involves determining whether the THV frame extends above the coronary ostia or sinotubular junction, as a VTC or VTSTJ distance of less than 2 mm has been associated with an increased risk of impaired coronary engagement. Additionally, an overlap between the THV sealing skirt and the coronary ostia is considered a significant predictor of difficult coronary reaccess. Another important factor is the spatial relationship between the commissural posts of the THV and the coronary ostia, as commissural misalignment may further hinder catheter manipulation. Multiplanar reconstructions of post-TAVR CCTA enable precise evaluation of commissural alignment, facilitating the creation of a short-axis THV view for anatomical assessment. Severe coronary overlap is defined when a THV commissure is positioned within 0–20° of the center of coronary ostium, whereas severe commissural misalignment is characterized by a deviation of more than 45° from the native commissure alignment [71].

## 17. Limitations

Despite its critical role in TAVR planning, CCTA has certain limitations that must be considered in clinical practice. One of the primary concerns is contrast-induced nephropathy, particularly in patients with pre-existing renal insufficiency. The use of iodinated contrast agents in CCTA poses a risk of worsening renal function, necessitating careful patient selection and optimization strategies such as pre-procedural hydration. Additionally, radiation exposure remains a consideration, although advancements in CT technology, including iterative reconstruction algorithms and dose reduction protocols, have significantly mitigated this risk. Furthermore, CCTA provides limited hemodynamic information compared to echocardiography and invasive hemodynamic assessment, making it less suitable for functional evaluation of aortic stenosis severity. Understanding these limitations is essential for optimizing the use of CCTA in TAVR workflows and ensuring its appropriate integration into patient evaluation and procedural planning.

## 18. Conclusions

CCTA plays a pivotal role in both the pre- and post-operative evaluation of patients undergoing TAVR. It has become the gold standard imaging modality for pre-procedural planning in native aortic stenosis as well as in ViV TAVR cases. Post-procedurally, CCTA remains essential for patient follow-up, allowing for the evaluation of THV expansion and coronary access and the detection of potential complications such as HALT, and structural valve degeneration.

## Abbreviations

CCTA	cardiac computed tomography angiography
HALT	hypoattenuating leaflet thickening
SHV	surgical heart valve
TAVR	transcatheter aortic valve replacement
THV	transcatheter heart valve
ViV	valve-in-valve
VTC	virtual THV-to-coronary distance
VTSTJ	virtual THV-to-sinotubular junction distance
